# Supplementary material for: A Likelihood Approach for Real-Time Calibration of Stochastic Compartmental Epidemic Models
Source: PLoS Comput Biol. 2017 Jan 17;13(1):e1005257. doi: 10.1371/journal.pcbi.1005257 (PMC5240920; doi:10.1371/journal.pcbi.1005257)
Supplement: S1 File — (TAR.GZ) [file pcbi.1005257.s014.tar.gz › HSPH_Online-SI-Revision/PF08/n100-severe/n100-severe_PF2_table-8.pdf]

1	newly 3 weeks spec	2276.	1148.96	{855., 1372.}	49.5185	0.303828	newly 3 weeks cumul	4764.	2709.93	{2073., 3203.}	43.1165	0.251296	newly 5 weeks spec	4385.	1834.21	{1379., 2220.}	58.1708	0.386753	newly 5 weeks cumul	12 438.	6001.7	{4519., 7199.}	51.7471	0.323516	newly 58 weeks cumul	64 638.	71 389.3	{62 803., 76 425.}	11.8612	0.0482918
2	newly 3 weeks spec	951.	489.48	{173., 824.}	59.8149	0.427699	newly 3 weeks cumul	1661.	991.29	{433., 1679.}	52.5557	0.343678	newly 5 weeks spec	4438.	1204.9	{295., 2070.}	76.0059	0.743345	newly 5 weeks cumul	8385.	2977.54	{1024., 5163.}	69.8421	0.591165	newly 65 weeks cumul	54 065.	75 243.8	{57 584., 90 887.}	39.7497	0.139792
3	newly 3 weeks spec	788.	380.2	{82., 755.}	66.9264	0.546545	newly 3 weeks cumul	1184.	733.91	{198., 1372.}	58.2779	0.407894	newly 5 weeks spec	4947.	1132.8	{125., 2508.}	83.1013	0.983905	newly 5 weeks cumul	8233.	2530.4	{461., 5247.}	76.9989	0.771769	newly 66 weeks cumul	68 234.	71 515.3	{47 679., 92 894.}	23.5747	0.102583
4	newly 3 weeks spec	1410.	607.04	{223., 1058.}	58.4567	0.450386	newly 3 weeks cumul	2503.	1265.31	{542., 2110.}	51.1622	0.3621	newly 5 weeks spec	4326.	1393.43	{339., 2797.}	69.0252	0.632291	newly 5 weeks cumul	9460.	3589.19	{1137., 6541.}	63.4079	0.521618	newly 64 weeks cumul	66 493.	72 467.5	{54 924., 90 592.}	19.4945	0.0789167
5	newly 3 weeks spec	2748.	1965.14	{1432., 2493.}	28.7453	0.1551	newly 3 weeks cumul	5863.	4549.73	{3462., 5623.}	23.217	0.120157	newly 5 weeks spec	4410.	3136.42	{2168., 4109.}	29.4	0.161496	newly 5 weeks cumul	13 943.	10 204.6	{7360., 13 005.}	27.2938	0.146326	newly 58 weeks cumul	66 068.	72 618.4	{63 373., 80 639.}	11.4759	0.04662
6	newly 3 weeks spec	1016.	491.89	{128., 1097.}	61.6644	0.480847	newly 3 weeks cumul	1815.	1019.59	{318., 2110.}	55.184	0.38902	newly 5 weeks spec	3575.	1159.45	{218., 2923.}	73.0867	0.720149	newly 5 weeks cumul	7380.	2943.72	{711., 6849.}	66.7431	0.576762	newly 64 weeks cumul	66 876.	72 444.2	{57 925., 90 744.}	17.1377	0.0694507
7	newly 3 weeks spec	815.	427.68	{140., 817.}	53.2368	0.39091	newly 3 weeks cumul	1311.	880.95	{348., 1645.}	44.3638	0.283084	newly 5 weeks spec	3565.	1026.52	{225., 2029.}	72.0022	0.693578	newly 5 weeks cumul	6614.	2578.93	{751., 4981.}	63.0945	0.524627	newly 65 weeks cumul	69 295.	72 971.7	{56 024., 90 830.}	17.6996	0.0739798
8	newly 3 weeks spec	1680.	876.81	{493., 1346.}	47.8125	0.308682	newly 3 weeks cumul	3165.	1956.16	{1227., 2842.}	38.9763	0.232867	newly 5 weeks spec	3946.	1624.11	{761., 2765.}	58.8416	0.425357	newly 5 weeks cumul	9677.	4777.94	{2644., 7538.}	50.6258	0.336105	newly 61 weeks cumul	59 021.	73 075.2	{60 525., 82 333.}	24.8049	0.094405
9	newly 3 weeks spec	240.	338.04	{83., 749.}	79.825	0.280792	newly 3 weeks cumul	369.	675.82	{208., 1373.}	105.111	0.292466	newly 5 weeks spec	1645.	888.86	{118., 2283.}	70.7818	0.575029	newly 5 weeks cumul	2651.	2111.95	{440., 4914.}	65.1034	0.388627	newly 66 weeks cumul	62 553.	70 561.3	{42 453., 92 070.}	28.5014	0.116812
10	newly 3 weeks spec	1249.	642.	{530., 766.}	48.5989	0.29353	newly 3 weeks cumul	2193.	1490.55	{1252., 1744.}	32.0315	0.171146	newly 5 weeks spec	4136.	1082.68	{868., 1307.}	73.823	0.587108	newly 5 weeks cumul	8808.	3407.51	{2842., 4041.}	61.3135	0.416376	newly 59 weeks cumul	62 587.	74 402.1	{72 335., 76 368.}	18.8779	0.074922
11	newly 3 weeks spec	804.	424.15	{110., 915.}	65.9938	0.498234	newly 3 weeks cumul	1294.	857.96	{275., 1728.}	58.2226	0.37349	newly 5 weeks spec	3948.	1049.81	{153., 3060.}	78.6563	0.875508	newly 5 weeks cumul	7140.	2582.77	{575., 6405.}	72.701	0.671902	newly 65 weeks cumul	63 055.	65 780.4	{42 495., 91 110.}	26.2487	0.113789
12	newly 3 weeks spec	2111.	977.41	{806., 1188.}	53.6992	0.338861	newly 3 weeks cumul	4254.	2292.48	{1920., 2694.}	46.11	0.272022	newly 5 weeks spec	4443.	1607.94	{1335., 1924.}	63.8096	0.446647	newly 5 weeks cumul	11 913.	5169.68	{4295., 6183.}	56.6047	0.366726	newly 58 weeks cumul	66 884.	74 014.7	{69 444., 76 525.}	10.6732	0.0437862
13	newly 3 weeks spec	2345.	1575.42	{1127., 2117.}	33.0277	0.186311	newly 3 weeks cumul	5116.	3664.15	{2752., 4944.}	28.7711	0.157171	newly 5 weeks spec	4572.	2516.65	{1753., 3458.}	44.9552	0.273925	newly 5 weeks cumul	13 208.	8194.22	{5955., 11 174.}	37.9602	0.21973	newly 58 weeks cumul	66 392.	73 344.7	{65 309., 79 816.}	11.9726	0.0487834
14	newly 3 weeks spec	1956.	1184.96	{912., 1432.}	39.4192	0.225164	newly 3 weeks cumul	4187.	2798.66	{2166., 3417.}	33.2013	0.181848	newly 5 weeks spec	3466.	1887.51	{1377., 2311.}	45.5421	0.273931	newly 5 weeks cumul	10 276.	6200.48	{4715., 7609.}	39.6606	0.227302	newly 58 weeks cumul	61 739.	72 394.4	{66 077., 77 175.}	17.529	0.069446
15	newly 3 weeks spec	1396.	535.43	{436., 637.}	61.6454	0.422547	newly 3 weeks cumul	2586.	1255.17	{1040., 1438.}	51.4629	0.319063	newly 5 weeks spec	3233.	906.82	{689., 1112.}	71.9511	0.560235	newly 5 weeks cumul	7869.	2860.99	{2328., 3376.}	63.6423	0.445589	newly 60 weeks cumul	52 704.	73 401.3	{67 980., 76 486.}	39.2708	0.143388
16	newly 3 weeks spec	1498.	762.47	{609., 945.}	49.1008	0.301602	newly 3 weeks cumul	2968.	1781.54	{1460., 2249.}	40.0081	0.228903	newly 5 weeks spec	3246.	1279.37	{1011., 1628.}	60.5863	0.413772	newly 5 weeks cumul	8486.	4056.24	{3303., 5140.}	52.2008	0.3286	newly 59 weeks cumul	63 449.	73 986.6	{69 714., 76 369.}	16.6245	0.0665074
17	newly 3 weeks spec	711.	562.62	{168., 1491.}	55.1336	0.31851	newly 3 weeks cumul	1220.	1110.39	{407., 2650.}	49.4336	0.248765	newly 5 weeks spec	2605.	1421.47	{266., 4406.}	66.4764	0.513198	newly 5 weeks cumul	5099.	3437.95	{936., 9624.}	60.0818	0.391548	newly 65 weeks cumul	53 195.	76 855.7	{57 474., 91 191.}	44.9643	0.155441
18	newly 3 weeks spec	1434.	600.28	{198., 1345.}	59.6095	0.472884	newly 3 weeks cumul	2751.	1286.96	{527., 2647.}	54.9364	0.40625	newly 5 weeks spec	4058.	1263.18	{351., 3572.}	68.8719	0.64489	newly 5 weeks cumul	9240.	3424.55	{1154., 8603.}	63.2841	0.534537	newly 63 weeks cumul	55 677.	71 054.7	{56 527., 89 827.}	28.6218	0.104313
19	newly 3 weeks spec	1846.	508.91	{226., 1052.}	72.4317	0.633184	newly 3 weeks cumul	3207.	1110.22	{557., 2211.}	65.4674	0.519728	newly 5 weeks spec	5657.	1038.12	{349., 2321.}	81.6489	0.85281	newly 5 weeks cumul	12 216.	2881.26	{1157., 5902.}	76.414	0.712825	newly 63 weeks cumul	62 777.	71 608.	{56 695., 85 819.}	18.0497	0.0708807
20	newly 3 weeks spec	1317.	460.89	{309., 779.}	65.0046	0.479671	newly 3 weeks cumul	2412.	1062.6	{751., 1713.}	55.9801	0.37662	newly 5 weeks spec	4511.	806.77	{554., 1409.}	82.1155	0.776941	newly 5 weeks cumul	9394.	2480.99	{1706., 4150.}	73.5896	0.602565	newly 61 weeks cumul	61 521.	74 032.6	{69 900., 76 826.}	20.3884	0.0801599
21	newly 3 weeks spec	780.	394.24	{110., 620.}	57.9	0.424879	newly 3 weeks cumul	1243.	810.99	{264., 1247.}	47.6227	0.304778	newly 5 weeks spec	3321.	949.47	{200., 1674.}	73.7769	0.717303	newly 5 weeks cumul	6292.	2377.1	{625., 4042.}	66.5782	0.557957	newly 65 weeks cumul	63 478.	74 848.1	{57 960., 90 908.}	21.9615	0.0840047
22	newly 3 weeks spec	720.	441.1	{178., 612.}	52.0083	0.325049	newly 3 weeks cumul	1304.	966.7	{435., 1329.}	41.6518	0.233352	newly 5 weeks spec	2369.	882.87	{278., 1348.}	69.9109	0.569941	newly 5 weeks cumul	4863.	2474.1	{928., 3626.}	59.914	0.412306	newly 63 weeks cumul	55 662.	70 915.1	{59 190., 80 414.}	28.4653	0.105812
23	newly 3 weeks spec	325.	727.14	{87., 1528.}	185.606	0.372916	newly 3 weeks cumul	484.	1205.77	{209., 2550.}	184.295	0.310517	newly 5 weeks spec	3196.	2128.16	{148., 4744.}	105.539	0.827248	newly 5 weeks cumul	4636.	4745.38	{455., 10 054.}	118.869	0.605259	newly 67 weeks cumul	66 380.	70 429.	{44 083., 92 758.}	23.2294	0.099709
24	newly 3 weeks spec	356.	538.89	{22., 1184.}	145.756	0.695806	newly 3 weeks cumul	524.	897.09	{35., 1949.}	139.292	0.576669	newly 5 weeks spec	2825.	1887.6	{24., 5827.}	106.995	1.10808	newly 5 weeks cumul	4283.	3902.2	{95., 10 550.}	114.228	0.893094	newly 68 weeks cumul	68 010.	60 929.4	{15 133., 89 885.}	34.8683	0.423473
25	newly 3 weeks spec	331.	538.01	{14., 1184.}	154.414	0.705653	newly 3 weeks cumul	441.	891.92	{32., 1949.}	165.687	0.592878	newly 5 weeks spec	2373.	1888.42	{20., 5827.}	115.089	1.0754	newly 5 weeks cumul	3717.	3897.61	{66., 10 550.}	123.286	0.879797	newly 68 weeks cumul	58 819.	61 596.3	{23 478., 89 885.}	41.5928	0.422021
26	newly 3 weeks spec	1190.	346.24	{218., 568.}	70.9042	0.569181	newly 3 weeks cumul	2010.	795.15	{526., 1267.}	60.4403	0.431124	newly 5 weeks spec	4173.	607.17	{354., 1090.}	85.45	0.881573	newly 5 weeks cumul	8492.	1871.08	{1185., 3153.}	77.9666	0.692056	newly 62 weeks cumul	69 666.	72 343.6	{65 766., 75 627.}	6.08948	0.0258763
27	newly 3 weeks spec	974.	372.18	{185., 579.}	63.1355	0.474681	newly 3 weeks cumul	1673.	829.24	{440., 1221.}	52.4734	0.353509	newly 5 weeks spec	3834.	718.04	{301., 1271.}	81.2718	0.808949	newly 5 weeks cumul	7556.	2066.48	{980., 3340.}	73.0204	0.624082	newly 63 weeks cumul	62 217.	73 488.7	{63 992., 82 580.}	18.767	0.0733112
28	newly 3 weeks spec	2141.	1586.82	{573., 2461.}	34.0084	0.217987	newly 3 weeks cumul	4227.	3274.26	{1373., 4858.}	30.5555	0.186003	newly 5 weeks spec	4410.	3303.14	{875., 5366.}	38.7868	0.259764	newly 5 weeks cumul	11 858.	8938.62	{2960., 13 945.}	34.9808	0.225396	newly 62 weeks cumul	54 529.	76 807.	{56 012., 90 378.}	41.5507	0.144132
29	newly 3 weeks spec	1545.	455.27	{173., 714.}	70.5327	0.592739	newly 3 weeks cumul	2666.	998.49	{405., 1524.}	62.5525	0.476107	newly 5 weeks spec	5090.	900.45	{306., 1503.}	82.3094	0.848365	newly 5 weeks cumul	10 609.	2538.46	{897., 4022.}	76.0726	0.691824	newly 63 weeks cumul	65 892.	71 538.5	{58 429., 80 653.}	14.135	0.0578715
30	newly 3 weeks spec	1626.	643.24	{517., 769.}	60.4403	0.407763	newly 3 weeks cumul	3106.	1513.09	{1249., 1845.}	51.2849	0.316494	newly 5 weeks spec	3975.	1064.08	{808., 1325.}	73.2307	0.578349	newly 5 weeks cumul	9727.	3410.93	{2759., 4064.}	64.9334	0.459897	newly 59 weeks cumul	54 604.	73 228.7	{70 461., 75 390.}	34.1086	0.127276
31	newly 3 weeks spec	312.	541.01	{14., 1184.}	162.343	0.697263	newly 3 weeks cumul	454.	895.9	{32., 1949.}	162.062	0.595211	newly 5 weeks spec	3043.	1904.83	{20., 5827.}	103.663	1.14275	newly 5 weeks cumul	4393.	3926.84	{66., 10 550.}	113.092	0.920632	newly 68 weeks cumul	60 937.	61 639.2	{23 478., 89 885.}	39.459	0.418892
32	newly 3 weeks spec	434.	357.1	{76., 764.}	66.8664	0.391095	newly 3 weeks cumul	710.	696.72	{188., 1384.}	62.0901	0.30627	newly 5 weeks spec	2775.	1033.17	{136., 2520.}	75.813	0.763778	newly 5 weeks cumul	4597.	2346.87	{443., 5365.}	69.7231	0.569919	newly 66 weeks cumul	62 201.	72 167.6	{47 563., 92 717.}	27.5525	0.109663
33	newly 3 weeks spec	975.	404.45	{83., 824.}	69.8246	0.601804	newly 3 weeks cumul	1516.	777.52	{196., 1629.}	62.2968	0.471707	newly 5 weeks spec	5236.	1176.33	{120., 2677.}	82.7859	0.979686	newly 5 weeks cumul	8992.	2656.9	{445., 5607.}	76.8817	0.779508	newly 66 weeks cumul	68 723.	70 132.5	{42 859., 93 165.}	25.1409	0.112984
34	newly 3 weeks spec	1779.	695.46	{569., 840.}	60.9073	0.412999	newly 3 weeks cumul	3127.	1644.27	{1371., 1949.}	47.417	0.283506	newly 5 weeks spec	4645.	1133.5	{916., 1401.}	75.5974	0.6184	newly 5 weeks cumul	10 816.	3670.87	{3078., 4443.}	66.0607	0.47415	newly 58 weeks cumul	64 121.	72 757.6	{71 098., 74 365.}	13.4692	0.054788
35	newly 3 weeks spec	295.	783.44	{81., 1538.}	221.708	0.387134	newly 3 weeks cumul	455.	1279.52	{192., 2592.}	215.574	0.33606	newly 5 weeks spec	2408.	2288.4	{152., 7061.}	118.762	0.730913	newly 5 weeks cumul	3628.	5059.49	{464., 13 085.}	140.911	0.553406	newly 67 weeks cumul	61 775.	75 176.6	{51 082., 93 395.}	30.1215	0.115677
36	newly 3 weeks spec	489.	416.58	{133., 930.}	52.135	0.291373	newly 3 weeks cumul	783.	832.79	{320., 1651.}	51.8914	0.229028	newly 5 weeks spec	2506.	1098.11	{195., 2932.}	64.8831	0.571296	newly 5 weeks cumul	4350.	2616.16	{682., 6345.}	57.0869	0.411325	newly 65 weeks cumul	66 421.	77 292.2	{58 701., 91 427.}	21.4556	0.0834115
37	newly 3 weeks spec	391.	535.53	{127., 1123.}	87.2097	0.306939	newly 3 weeks cumul	633.	1019.92	{272., 2121.}	92.5782	0.273715	newly 5 weeks spec	3280.	1542.56	{214., 3894.}	72.7768	0.649921	newly 5 weeks cumul	5202.	3498.41	{708., 7782.}	68.8449	0.471144	newly 67 weeks cumul	56 370.	71 621.9	{45 650., 92 383.}	36.1168	0.134327
38	newly 3 weeks spec	728.	447.15	{121., 631.}	63.3558	0.409884	newly 3 weeks cumul	1154.	880.1	{310., 1169.}	52.6153	0.289631	newly 5 weeks spec	4350.	1137.44	{208., 1721.}	81.0069	0.827448	newly 5 weeks cumul	7337.	2747.9	{682., 3872.}	74.8606	0.628283	newly 65 weeks cumul	69 743.	75 519.7	{55 940., 91 488.}	18.4029	0.0758851
39	newly 3 weeks spec	1971.	1033.83	{551., 1828.}	54.0492	0.363039	newly 3 weeks cumul	3791.	2211.96	{1315., 3745.}	48.2269	0.301684	newly 5 weeks spec	4327.	2077.35	{882., 3624.}	59.8606	0.435871	newly 5 weeks cumul	11 151.	5782.76	{2908., 9961.}	55.4262	0.377306	newly 62 weeks cumul	53 564.	73 516.9	{63 460., 85 994.}	37.2505	0.134129
40	newly 3 weeks spec	1025.	800.4	{183., 1622.}	65.4927	0.390274	newly 3 weeks cumul	1775.	1555.63	{461., 2940.}	58.6642	0.309576	newly 5 weeks spec	3528.	2062.89	{268., 4443.}	72.6715	0.583777	newly 5 weeks cumul	7401.	4956.06	{1022., 10 015.}	68.0073	0.46714	newly 65 weeks cumul	51 673.	68 938.3	{42 251., 92 701.}	41.7252	0.148428
41	newly 3 weeks spec	676.	1168.28	{113., 1515.}	163.405	0.485467	newly 3 weeks cumul	1025.	1829.78	{276., 2535.}	149.321	0.387745	newly 5 weeks spec	4452.	2795.64	{154., 6409.}	98.6208	0.813442	newly 5 weeks cumul	7242.	6770.26	{599., 11 764.}	113.557	0.649813	newly 67 weeks cumul	64 974.	74 551.9	{47 995., 92 585.}	26.7164	0.108663
42	newly 3 weeks spec	1593.	552.87	{428., 720.}	65.2938	0.46867	newly 3 weeks cumul	2901.	1283.64	{989., 1628.}	55.7518	0.362118	newly 5 weeks spec	4138.	951.02	{692., 1265.}	77.0174	0.650691	newly 5 weeks cumul	9608.	2960.29	{2237., 3903.}	69.1893	0.520789	newly 60 weeks cumul	61 993.	74 275.6	{69 527., 77 221.}	19.8129	0.078135
43	newly 3 weeks spec	1692.	840.87	{615., 1042.}	50.3032	0.313973	newly 3 weeks cumul	3552.	1964.91	{1510., 2475.}	44.7396	0.266155	newly 5 weeks spec	3812.	1413.67	{997., 1787.}	62.9153	0.443067	newly 5 weeks cumul	9984.	4472.97	{3269., 5697.}	55.1986	0.358903	newly 59 weeks cumul	48 969.	73 791.6	{68 123., 77 016.}	50.6905	0.17753
44	newly 3 weeks spec	459.	406.59	{93., 772.}	67.8671	0.373456	newly 3 weeks cumul	732.	786.2	{222., 1475.}	63.7896	0.287577	newly 5 weeks spec	2554.	1203.72	{167., 2598.}	73.4699	0.674128	newly 5 weeks cumul	4392.	2698.3	{544., 5455.}	67.3138	0.500977	newly 66 weeks cumul	67 035.	71 089.9	{46 230., 92 336.}	24.6858	0.106566
45	newly 3 weeks spec	956.	480.05	{134., 938.}	54.2437	0.417643	newly 3 weeks cumul	1721.	1003.92	{308., 1842.}	48.2824	0.339806	newly 5 weeks spec	3513.	1088.74	{211., 2489.}	70.7714	0.685015	newly 5 weeks cumul	7079.	2821.59	{685., 5920.}	62.7415	0.533543	newly 64 weeks cumul	64 288.	73 341.6	{57 844., 90 724.}	18.8804	0.0732768
46	newly 3 weeks spec	720.	350.7	{163., 586.}	51.9472	0.366809	newly 3 weeks cumul	1225.	779.22	{400., 1189.}	39.5396	0.248386	newly 5 weeks spec	3211.	680.75	{280., 1291.}	78.7994	0.748625	newly 5 weeks cumul	5975.	1953.03	{877., 3440.}	67.3133	0.541259	newly 63 weeks cumul	66 403.	74 192.6	{66 267., 82 209.}	12.4695	0.050145
47	newly 3 weeks spec	2512.	1887.79	{1411., 2309.}	25.3587	0.135288	newly 3 weeks cumul	5499.	4245.76	{3298., 5081.}	23.3933	0.122013	newly 5 weeks spec	4726.	3257.23	{2252., 4144.}	31.0954	0.173619	newly 5 weeks cumul	13 753.	10 026.4	{7460., 12 391.}	27.3531	0.147683	newly 59 weeks cumul	63 771.	77 617.5	{69 241., 83 597.}	21.8805	0.0849334
48	newly 3 weeks spec	1312.	604.77	{186., 1094.}	59.2508	0.460415	newly 3 weeks cumul	2348.	1249.78	{449., 2110.}	52.8245	0.37464	newly 5 weeks spec	4095.	1400.25	{321., 2842.}	69.5885	0.649891	newly 5 weeks cumul	8888.	3583.19	{1011., 6683.}	64.2002	0.535007	newly 64 weeks cumul	66 105.	73 848.8	{57 753., 91 203.}	19.3458	0.0763384
49	newly 3 weeks spec	197.	537.29	{22., 1184.}	236.381	0.619101	newly 3 weeks cumul	276.	892.11	{35., 1949.}	263.149	0.580916	newly 5 weeks spec	2370.	1886.34	{24., 5827.}	115.241	1.06514	newly 5 weeks cumul	3259.	3894.53	{95., 10 550.}	133.209	0.835899	newly 68 weeks cumul	66 569.	61 137.9	{22 643., 89 885.}	35.501	0.420061
50	newly 3 weeks spec	580.	538.54	{104., 1072.}	82.7069	0.418929	newly 3 weeks cumul	912.	966.37	{238., 2066.}	74.909	0.323176	newly 5 weeks spec	4132.	1659.89	{161., 3370.}	84.6038	0.82857	newly 5 weeks cumul	6745.	3634.62	{546., 7211.}	81.3067	0.637692	newly 67 weeks cumul	62 718.	71 869.9	{48 491., 92 692.}	27.4225	0.11153
